# Serum Gamma - Glutamyltransferase Is Associated with Albuminuria: A Population-Based Study

**DOI:** 10.1371/journal.pone.0114970

**Published:** 2014-12-11

**Authors:** Kan Sun, Feng Li, Diaozhu Lin, Yiqin Qi, Mingtong Xu, Na Li, Chulin Huang, Meng Ren, Yan Li, Li Yan

**Affiliations:** Department of Endocrinology, Sun Yat-sen Memorial Hospital, Sun Yat-sen University, 107 Yanjiang West Road, Guangzhou, 510120, People' s Republic of China; School of Public Health of University of São Paulo, Brazil

## Abstract

**Background:**

Serum γ - glutamyltransferase (GGT) is implicated in the pathogenesis of endothelial dysfunction and atherosclerosis. Albuminuria is a marker of endothelial damage and correlated with structural and functional integrity of the vasculature. Our objective was to evaluate the association between serum GGT level and prevalence of albuminuria in a Chinese population.

**Materials and Methods:**

We conducted a population-based cross-sectional study in 9,702 subjects aged 40 years or older. Increased urinary albumin excretion was defined according to the urinary albumin-to-creatinine ratio (ACR) ranges greater or equal than 30 mg/g. Low-grade albuminuria was defined according to the highest quartile of ACR in participants without increased urinary albumin excretion.

**Results:**

The prevalence of low-grade albuminuria and increased urinary albumin excretion were respectively 23.4% and 6.6% in this population and gradually increased across the sex-specific serum GGT quartiles (all P for trend <0.05). In logistic regression analysis, compared with subjects in the lowest quartile of serum GGT level, the adjusted odds ratios (ORs) in the highest quartile was 1.22 [95% confidence interval (CI), 1.04–1.43] for low-grade albuminuria and 1.55 (95% CI, 1.18–2.04) for increased urinary albumin excretion. In subgroup analysis, significant relationship of serum GGT level with both low-grade albuminuria and increased urinary albumin excretion were detected in women, younger subjects, overweight subjects and in those with hypertension or glomerular filtration rate greater than 90 (all P <0.05).

**Conclusion:**

Serum GGT level is associated with urinary albumin excretion in middle-aged and elderly Chinese.

## Introduction

Serum γ - glutamyltransferase (GGT) is traditionally used as a biological marker for excessive alcohol consumption or liver diseases [1]. Evidence has shown that GGT is related to oxidative stress and endothelial dysfunction which might therefore have a role in the pathogenesis of other diseases [2], [3], [4]. Recent prospective studies show that serum GGT is associated with atherosclerotic risk factors and could be a predictor of metabolic syndrome, diabetes, cardiovascular diseases and future mortality [5], [6], [7].

Albuminuria is thought to be a marker of endothelial dysfunction and even small levels of albuminuria could signify extensive endothelial damage [8], [9]. Increased urinary albumin excretion is including historical micro- and macro-albuminuria, which is defined as the urinary albumin-to-creatinine ratio (ACR) ranges greater or equal than 30 mg/g [10]. Studies conducted over the past decades have provided substantial evidence that increased urinary albumin excretion is a risk factor for diabetic nephropathy and cardiovascular diseases [8], [11], [12]. Recent studies indicated that low-grade albuminuria (ACR less than 30 mg/g) is associated with abnormal cardiac mechanics [13] and might also increase the risk of cardiovascular morbidity and mortality [14], [15].

We hypothesized that the influence of serum GGT level on the presence or progression of albuminuria is critical. However, less is known concerning the association between serum GGT level and albuminuria. To clarify such relationship would be conducive to the prevention and treatment of albuminuria and its related diseases. We therefore analyzed data from a Chinese population to explore the possible relationship between serum GGT level and albuminuria.

## Subjects and Methods

### Study population and design

We performed a cross-sectional study in a community, in Guangzhou, China, from June to November, 2011. Study population is from the Risk Evaluation of cAncers in Chinese diabeTic Individuals: A lONgitudinal (REACTION) study, which has been set up as a multicenter prospective observational study aiming to evaluate the chronic diseases in Chinese population [16], [17].

The study population, design, and protocols have been described previously [18]. Briefly, a total of 10,104 residents aged 40 years or older were invited to participate by examination notice or home visits. Totally, there were 9,916 subjects signed the consent form and agreed to participate in the survey, with a participation rate of 98.1%. Subjects who failed to provid information on urinary albumin and creatinine (n = 160) or serum GGT level (n = 54) were excluded from analysis. Accordingly, a total of 9,702 eligible individuals were included in the final data analyses. The study protocol was approved by the Institutional Review Board of the Sun Yat-sen Memorial Hospital affiliated to Sun Yat-sen University and was in accordance with the principle of the Helsinki Declaration II. Written informed consent was obtained from each participant before data collection.

### Clinical and biochemical measurements

We collected information on lifestyle factors, medical history, sociodemographic characteristics and family history by using a standard questionnaire. Smoking or drinking habit was classified as ‘never’, ‘current’ (smoking or drinking regularly in the past 6 months) or ‘ever’ (cessation of smoking or drinking more than 6 months) [19]. A short form of the International Physical Activity Questionnaire (IPAQ) was used to estimate physical activity at leisure time by adding questions on frequency and duration of moderate or vigorous activities and walking [20]. Separate metabolic equivalent hours per week (MET-h/week) were calculated for evaluation of total physical activity.

All participants completed anthropometrical measurements with the assistance of trained staff by using standard protocols. Three times consecutively blood pressure measurements by the same observer with a 5 minute interval were obtained by an automated electronic device (OMRON, Omron Company, China). The average of three measurements of blood pressure was used for analysis. Body height and body weight were recorded to the nearest 0.1 cm and 0.1 kg while participants were wearing light indoor clothing without shoes. Body mass index (BMI) was calculated as weight in kilograms divided by height in meters squared (kg/m^2^). Obesity was defined as BMI equal or greater than 28 and overweight was defined as BMI equal or greater than 24 and less than 28. Waist circumference (WC) was measured at the umbilical level with participant in standing position, at the end of gentle expiration.

Venous blood samples were collected for laboratory tests after an overnight fasting of at least 10 hours. Measurement of fasting plasma glucose (FPG), fasting serum insulin, triglycerides (TG), total cholesterol (TC), high-density lipoprotein cholesterol (HDL-C), low-density lipoprotein cholesterol (LDL-C), creatinine, GGT, aspartate aminotransferase (AST) and alanine aminotransferase (ALT) was done using an autoanalyser (Beckman CX-7 Biochemical Autoanalyser, Brea, CA, USA). Hemoglobin A1c (HbA1c) was assessed by high-performance liquid chromatography (Bio-Rad, Hercules, CA). The abbreviated Modification of Diet in Renal Disease (MDRD) formula recalibrated for Chinese population was used to calculate estimated glomerular filtration rate (GFR) expressed in mL/min per 1.73 m^2^ using a formula of eGFR  = 186 × [serum creatinine ×0.011]^−1.154^ × [age]^−0.203^ × [0.742 if female] ×1.233, where serum creatinine was expressed as µmol/L and 1.233 was the adjusting coefficient for Chinese population [21]. The insulin resistance index (homeostasis model assessment of insulin resistance, HOMA-IR) was calculated as fasting insulin ( µIU/ml) × fasting glucose (mmol/L)/22.5 [22]. Diabetes was diagnosed according to the 1999 World Health Organization diagnostic criteria.

### Definition of increased urinary albumin excretion and low-grade albuminuria

The first morning spot urine samples were collected for assessing the ACR. Urine albumin and creatinine were measured by chemiluminescence immunoassay (Siemens Immulite 2000, United States) and the Jaffe's kinetic method (Biobase-Crystal, Jinan, China) on the automatic analyzer, respectively. ACR was calculated by dividing the urinary albumin concentrations by the urinary creatinine concentrations and expressed in mg/g. Increased urinary albumin excretion was defined according to the ACR ranges greater or equal than 30 mg/g. The definition of low-grade albuminuria was according to the highest quartile of ACR in participants without increased urinary albumin excretion (ACR greater or equal than 11.12 mg/g and less than 30 mg/g in the present study).

### Statistical analysis

Statistical analysis was performed using SAS version 9.2 (SAS Institute Inc, Cary, NC, USA). Continuous variables were presented as means ± standard deviation (SD) except for skewed variables, which were presented as medians (interquartile ranges). Categorical variables were expressed as numbers (proportions). FPG, TG, HOMA-IR, GFR and MET-h/week were logarithmically transformed before analysis due to a non-normal distribution. Serum GGT level (U/L) was presented as quartiles: Quartile 1, <15; Quartile 2, 15 to <20; Quartile 3, 20 to <29; and Quartile 4, ≥29. Linear regression analysis was used to test for trend across groups. Differences among groups were tested by one-way ANOVA and *post hoc* comparisons were performed by using Bonferroni correction. Comparisons between categorical variables were performed with the χ^2^ test.

We analyzed the impact of serum GGT on the prevalence of low-grade albuminuria, increased urinary albumin excretion and decreased eGFR. The unadjusted and multivariate-adjusted logistic regression analysis was used to assess the risk of prevalent low-grade albuminuria, increased urinary albumin excretion and decreased eGFR in relation to each quartile increase in serum GGT level. Odds ratios (OR) and the corresponding 95% confidence intervals (95% CI) were calculated.

Relationship between serum GGT level and albuminuria was also explored in subgroups stratified by gender (men/women), age (≥60/<60 years), degree of obesity (normal/overweight/obesity), hypertension (yes/no), diabetes (yes/no) and GFR (>90/≤90). Tests for interaction were performed with including simultaneously each strata factor, the quartiles of serum GGT level and the respective interaction terms (strata factor multiplied by quartiles of serum GGT level) in the models.

All statistical tests were two-sided, and a P value <0.05 was considered statistically significant.

## Results

### Clinical characteristics of the study population

Among the 9702 enrolled individuals, the mean age was 55.9±8.1 years. The median serum GGT level was 20 U/L with interquartile range 15 to 29 U/L. Table 1 shows the characteristics of the participants according to urinary albumin excretion. There were 2267 (23.4%) participants categorized as low-grade albuminuria and 640 (6.6%) categorized as increased urinary albumin excretion, respectively. Compared with normal urinary albumin excretion subjects, those with low-grade albuminuria or increased urinary albumin excretion had significantly higher serum GGT level (all P<0.05).

**Table 1:** Characteristics of study population by urinary albumin excretion status.

	Normal urinary albumin excretion	Low-grade albuminuria	Increased urinary albumin excretion	P for trend
n (%)	6795 (70.0)	2267 (23.4)	640 (6.6)	
Urinary albumin to creatinine ratio (mg/g)	6.65 (5.02–8.40)	14.71 (12.53–18.99)*	53.13 (37.8–94.5)*	<0.0001
GGT (U/L)	19 (14–28)	21 (15–30)*	24 (16–35)*	<0.0001
Age (years)	55.5±7.7	56.7±8.4*	58.0±9.6*	<0.0001
Male [n (%)]	2097 (30.9)	501 (22.1)	190 (29.7)	<0.0001
BMI (kg/m^2^)	23.4±3.2	24.0±3.8*	24.7±3.7*	<0.0001
WC (cm)	81.1±9.2	82.3±9.9*	85.1±10.4*	<0.0001
SBP (mmHg)	124.2±15.6	128.9±16.8*	134.9±18.9*	<0.0001
DBP (mmHg)	74.6±9.5	76.4±10.4*	78.8±11.0*	<0.0001
Current smoker [n (%)]	702 (10.6)	163 (7.4)	74 (11.8)	0.070
Current drinker [n (%)]	227 (3.5)	67 (3.1)	23 (3.7)	0.777
TG (mg/dL)	108.85 (80.53–157.52)	115.93 (83.19–170.80)*	138.05 (97.35–194.69)*	<0.0001
TC (mg/dL)	200.39±46.72	204.25±47.88*	203.09±52.12	0.002
HDL-C (mg/dL)	51.35±13.90	51.35±13.51	47.88±12.74*	<0.0001
LDL-C (mg/dL)	121.24±35.91	122.78±37.45	122.78±38.61	0.083
FPG (mg/dL)	97.30 (90.09–105.95)	98.92 (90.81–109.00)*	100.72 (92.07–120.90)*	<0.0001
HbA1C (%)	5.95±0.73	6.17±1.05*	6.55±1.53*	<0.0001
HOMA-IR	1.67 (1.19–2.40)	1.88 (1.29–2.80)*	2.23 (1.45–3.48)*	<0.0001
eGFR (ml/min per 1.73 m^2^)	112.9±22.0	113.5±23.9	108.6±31.9*	0.006
Physical activity (MET-h/week)	21.0 (10.5–45.0)	21.0 (10.5–42.0)	21.5 (10.5–42.0)	0.758

1. Data were means ± SD or medians (interquartile ranges) for skewed variables or numbers (proportions) for categorical variables.

2. P for trend was calculated for the linear regression analysis tests across the groups. P values were for the ANOVA or χ2 analyses across the groups.

3. *P<0.05 compared with normal urinary albumin excretion group.

4. BMI, body mass index; WC, waist circumference; SBP, systolic blood pressure; DBP, diastolic blood pressure; TG, triglycerides; TC, total cholesterol; HDL-C, high-density lipoprotein cholesterol; LDL-C, low-density lipoprotein cholesterol; FPG, fasting plasma glucose; HOMA-IR, homeostasis model assessment of insulin resistance; eGFR, estimated glomerular filtration rate; GGT, γ-glutamyltransferase.

The clinical and biochemical characteristics according to the quartiles of serum GGT level were presented in Table 2. Participants with higher serum GGT level had elevated BMI, WC, SBP, DBP, TG, TC, LDL-C, FPG, HbA1C, AST, ALT, HOMA-IR and higher proportions of increased urinary albumin excretion, current smokers and current drinkers (all P for trend <0.0001).

**Table 2:** Characteristics of study population by γ-glutamyltransferase quartiles (U/L).

	Quartile 1 (<15)	Quartile 2 (15 to <20)	Quartile 3 (20 to <29)	Quartile 4 (≥29)	P for trend
n (%)	2423 (25.0)	2346 (24.2)	2500 (25.8)	2433 (25.1)	
GGT (U/L)	12 (10–13)	17 (16–18)*	23 (21–26)*	39 (33–54)*	<0.0001
Prevalence of low-grade albuminuria	542 (23.4)	500 (22.5)	620 (26.6)	605 (27.5)	<0.0001
Prevalence of increased urinary albumin excretion	111 (4.6)	128 (5.5)	165 (6.6)	236 (9.7)	<0.0001
Age (years)	54.1±8.0	55.9±8.0*	57.1±8.2*	56.6±7.7*	<0.0001
Male [n (%)]	281 (11.6)	524 (22.3)	862 (34.5)	1121 (46.1)	<0.0001
BMI (kg/m^2^)	22.8±3.1	23.3±3.3*	24.0±3.5*	24.5±3.4*	<0.0001
WC (cm)	78.2±9.0	80.4±9.1*	82.8±9.1*	85.3±9.3*	<0.0001
SBP (mmHg)	121.2±15.6	124.8±15.7*	127.8±16.6*	130.2±16.3*	<0.0001
DBP (mmHg)	72.7±9.5	74.8±9.6*	75.9±9.6*	77.8±10.1*	<0.0001
Current smoker [n (%)]	92 (3.9)	175 (7.7)	279 (11.4)	394 (16.7)	<0.0001
Current drinker [n (%)]	40 (1.7)	43 (1.9)	82 (3.4)	152 (6.5)	<0.0001
TG (mg/dL)	84.07 (64.60–111.50)	107.08 (81.42–146.02)*	127.43 (92.04–177.88)*	149.56 (103.54–214.16)*	<0.0001
TC (mg/dL)	177.61±49.42	203.09±41.70*	210.42±43.63*	214.29±45.17*	<0.0001
HDL-C (mg/dL)	49.81±15.44	53.28±13.51*	51.35±12.74*	49.81±12.74	0.035
LDL-C (mg/dL)	106.18±35.52	123.17±33.59*	127.80±35.91*	128.96±36.29*	<0.0001
FPG (mg/dL)	94.95 (88.29–102.34)	96.76 (89.73–104.86)*	98.92 (91.35–108.47)*	101.44 (92.79–113.51)*	<0.0001
HbA1C (%)	5.86±0.63	5.95±0.71*	6.08±0.90*	6.28±1.19*	<0.0001
HOMA-IR	1.40 (1.03–1.90)	1.63 (1.18–2.28)*	1.93 (1.33–2.73)*	2.23 (1.55–3.30)*	<0.0001
eGFR (ml/min per 1.73 m^2^)	121.7±30.0	111.2±19.0*	108.9±19.7*	109.4±19.9*	<0.0001
AST (U/L)	15 (12–18)	18 (15–21)*	19 (16–22)*	22 (18–27)*	<0.0001
ALT (U/L)	10 (7–13)	12 (9–15)*	13 (10–18)*	17 (13–25)*	<0.0001
Physical activity (MET-h/week)	21.0 (10.5–45.0)	24.0 (10.5–49.0)	21.0 (10.5–42.0)	21.0 (10.5–42.0)	0.308

1. Data were means ± SD or medians (interquartile ranges) for skewed variables or numbers (proportions) for categorical variables.

2. P for trend was calculated for the linear regression analysis tests across the groups. P values were for the ANOVA or χ2 analyses across the groups.

3. *P<0.05 compared with quartile 1 of GGT level.

4. BMI, body mass index; WC, waist circumference; SBP, systolic blood pressure; DBP, diastolic blood pressure; TG, triglycerides; TC, total cholesterol; HDL-C, high-density lipoprotein cholesterol; LDL-C, low-density lipoprotein cholesterol; FPG, fasting plasma glucose; HOMA-IR, homeostasis model assessment of insulin resistance; eGFR, estimated glomerular filtration rate; GGT, γ-glutamyltransferase.

### Associations of serum GGT level with albuminuria

**Figure 1:**
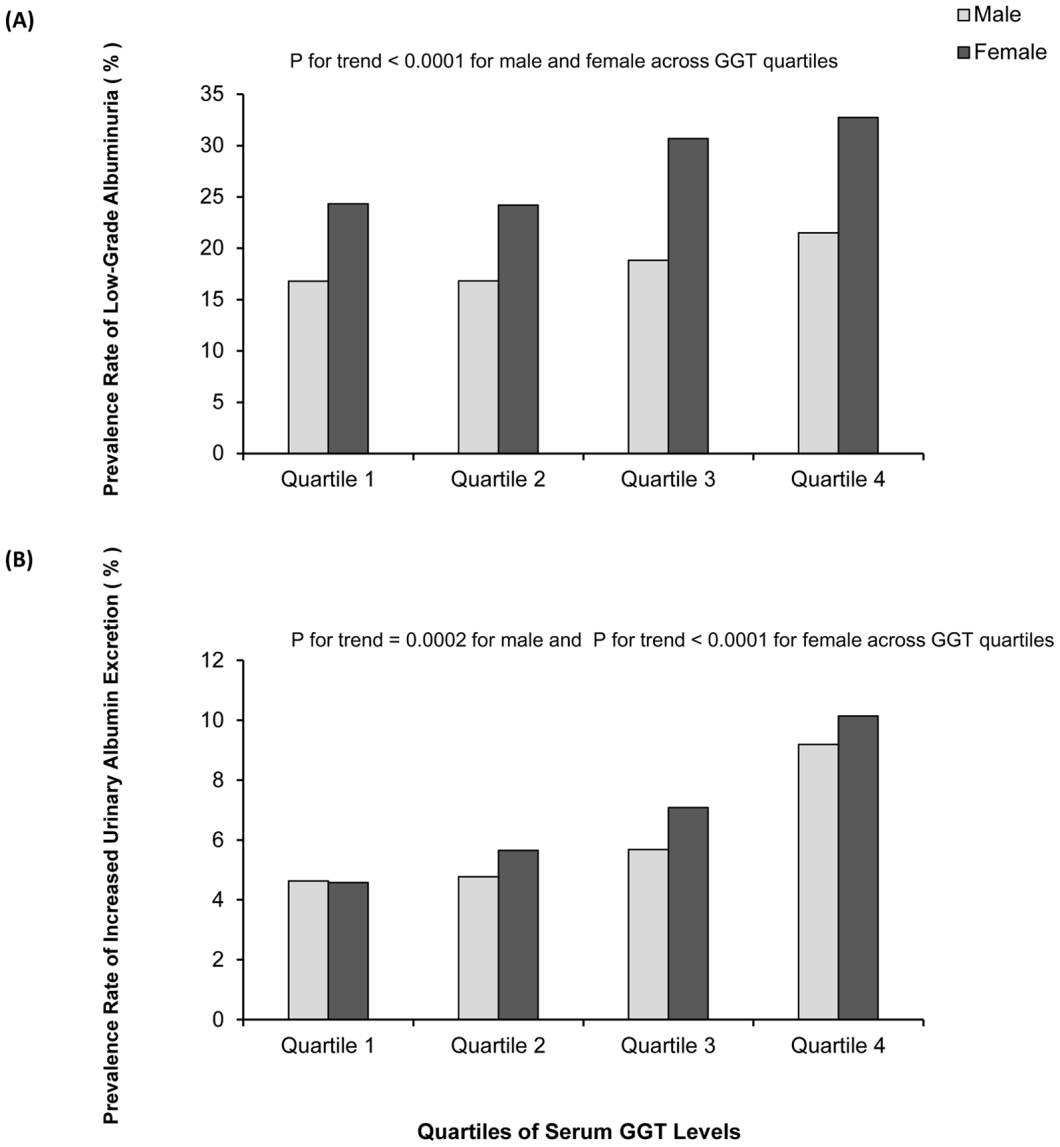
Prevalence of low-grade albuminuria and increased urinary albumin excretion in different quartiles of serum γ-glutamyltransferase (GGT) levels. (A) Low-grade albuminuria, (B) Increased urinary albumin excretion.

As shown in Fig. 1A, from the lowest quartile to the highest quartile of serum GGT level, the prevalence of low-grade albuminuria were 16.8%, 16.8%, 18.8%, 21.5% in male and 24.3%, 24.2%, 30.7%, 32.7% in female, respectively (all P for trend <0.0001). Strikingly, the prevalence of increased urinary albumin excretion tended to increase with the elevated serum GGT quartile (Fig. 1B 4.6%, 4.8%, 5.7% and 9.2% in male, P for trend  = 0.0002; 4.6%, 5.6%, 7.1% and 10.1% in female, P for trend <0.0001).

As shown in Table 2, compared with participants in quartile 1 of serum GGT, univariate logistic regression analysis showed that participants in quartile 3 and quartile 4, respectively, have a significant correlation with increased odds of low-grade albuminuria and increased urinary albumin excretion (all P for trend <0.0001). In multivariate logistic regression analyses (model 3), the ORs of low-grade albuminuria for increasing serum GGT quartiles were 1.00 (reference), 0.94 (95% CI 0.81–1.10), 1.15 (95% CI 0.99–1.34) and 1.22 (95% CI 1.04–1.43). Similarly, the ORs of increased urinary albumin excretion for increasing serum GGT quartiles in model 3 were 1.00 (reference), 1.03 (95% CI 0.78–1.36), 1.07 (95% CI 0.82–1.41) and 1.55 (95% CI 1.18–2.04), respectively (Table 3). Similar results were found in participants with eGFR <90 ml/min per 1.73 m^2^ but not in those with eGFR <60 ml/min per 1.73 m^2^ in the present study (Table 4).

**Table 3:** Prevalence of low-grade albuminuria and increased urinary albumin excretion according to quartiles of serum γ-glutamyltransferase levels.

		Quartile 1	Quartile 2	Quartile 3	Quartile 4	P for trend
Low-Grade albuminuria	Model 1	1	0.95 (0.83–1.09)	1.18 (1.03–1.35)	1.24 (1.09–1.42)	<0.0001
	Model 2	1	0.95 (0.83–1.10)	1.19 (1.03–1.37)	1.33 (1.15–1.54)	<0.0001
	Model 3	1	0.94 (0.81–1.10)	1.15 (0.99–1.34)	1.22 (1.04–1.43)	0.0024
Increased urinary albumin excretion	Model 1	1	1.20 (0.93–1.56)	1.47 (1.15–1.89)	2.24 (1.77–2.83)	<0.0001
	Model 2	1	1.11 (0.85–1.44)	1.26 (0.97–1.63)	1.98 (1.55–2.54)	<0.0001
	Model 3	1	1.03 (0.78–1.36)	1.07 (0.82–1.41)	1.55 (1.18–2.04)	0.0008

Data are odds ratios (95% confidence interval). Participants without low-grade albuminuria or increased urinary albumin excretion are defined as 0 and with low-grade albuminuria or increased urinary albumin excretion as 1.

Model 1 is unadjusted.

Model 2 is adjusted for age, sex and BMI.

Model 3 is adjusted for age, sex, BMI, current smoking and drinking status, SBP, TG, LDL-C, HOMA-IR, eGFR and physical activity.

**Table 4:** Prevalence of decreased eGFR according to quartiles of serum γ-glutamyltransferase levels.

		Quartile 1	Quartile 2	Quartile 3	Quartile 4	P for trend
Participants with eGFR <90 ml/min per 1.73 m^2^	Model 1	1	1.79 (1.44–2.22)	2.63 (2.15–3.23)	2.63 (2.14–3.23)	<0.0001
	Model 2	1	1.51 (1.20–1.90)	1.92 (1.54–2.39)	1.97 (1.58–2.46)	<0.0001
	Model 3	1	1.24 (0.98–1.58)	1.47 (1.16–1.85)	1.38 (1.09–1.76)	0.008
Participants with eGFR <60 ml/min per 1.73 m^2^	Model 1	1	0.93 (0.38–2.29)	2.63 (1.27–5.45)	2.10 (0.99–4.47)	0.007
	Model 2	1	0.83 (0.33–2.11)	1.79 (0.82–3.90)	1.54 (0.68–3.47)	0.112
	Model 3	1	0.52 (0.19–1.45)	1.25 (0.55–2.82)	0.95 (0.40–2.30)	0.600

Data are odds ratios (95% confidence interval). Participants without decreased eGFR are defined as 0 and with decreased eGFR as 1.

Model 1 is unadjusted.

Model 2 is adjusted for age, sex and BMI.

Model 3 is adjusted for age, sex, BMI, current smoking and drinking status, SBP, TG, LDL-C, HOMA-IR, ACR and physical activity.

### Serum GGT level and prevalence of albuminuria in different subgroups

**Figure 2:**
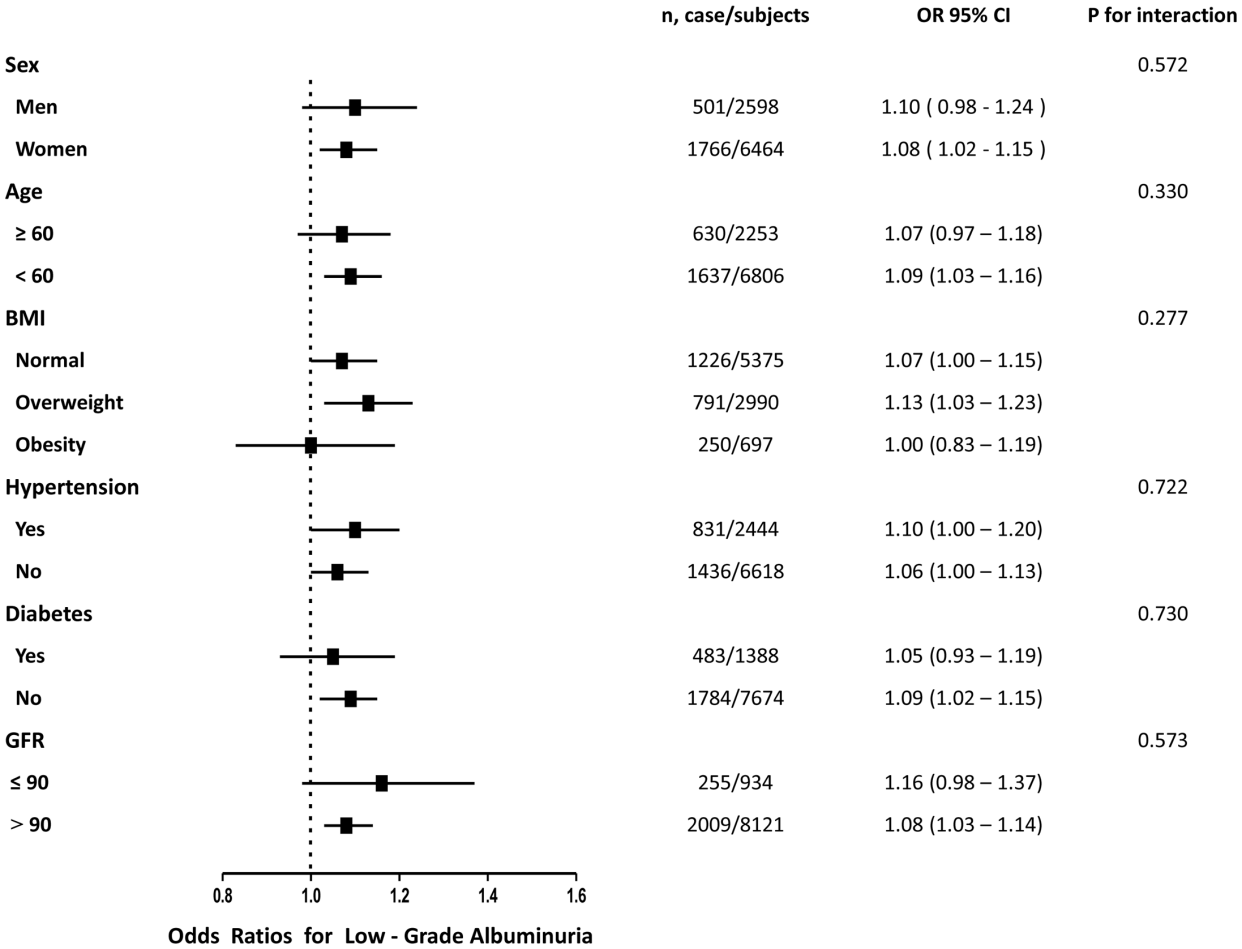
Prevalence of low-grade albuminuria with each quartile increase of serum γ-glutamyltransferase levels in different subgroups.

**Figure 3:**
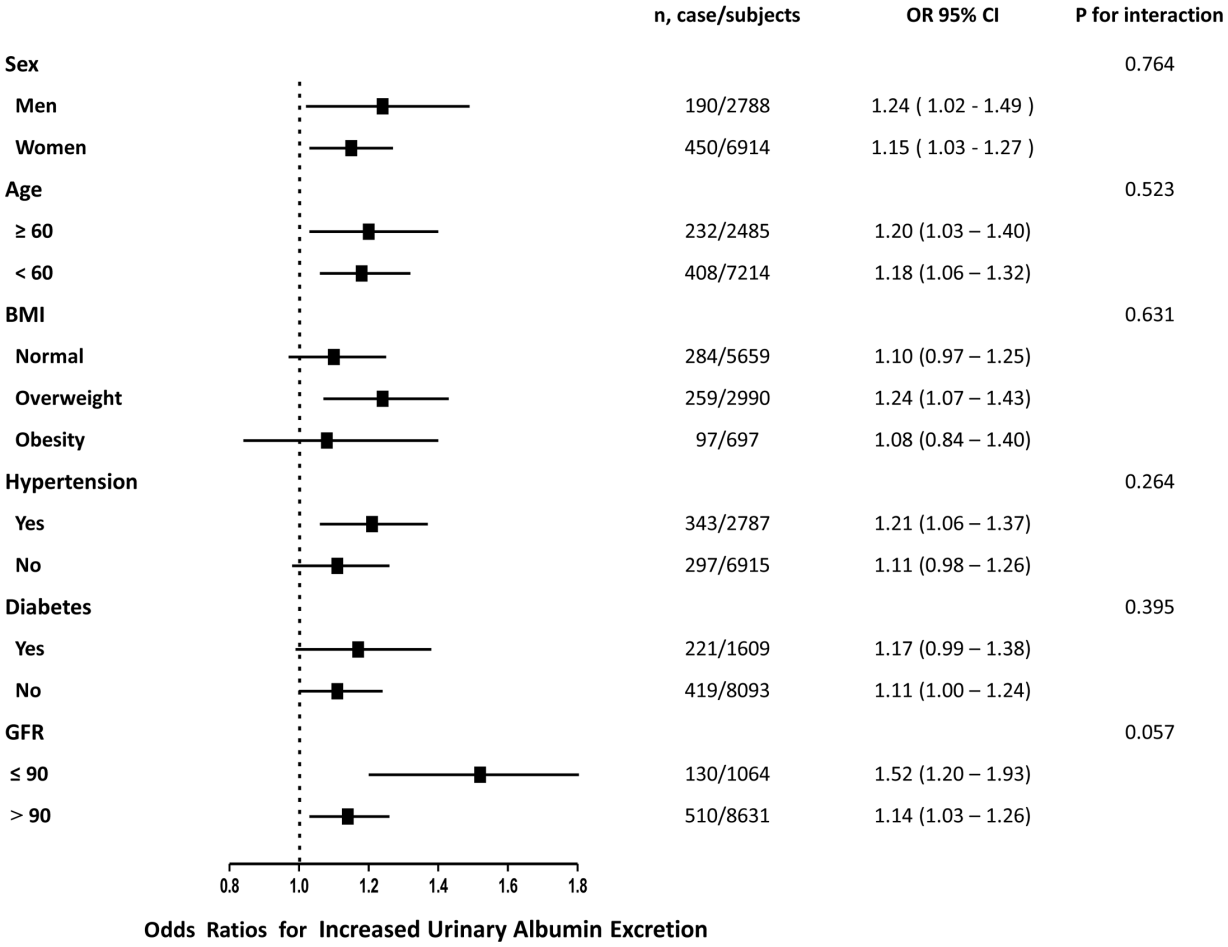
Prevalence of increased urinary albumin excretion with each quartile increase of serum γ-glutamyltransferase levels in different subgroups.

As shown in Figs. 2 and 3, the associations of serum GGT level with low-grade albuminuria and increased urinary albumin excretion were not consistently the same in subgroups analyses. Significant relationship of serum GGT level with both low-grade albuminuria and increased urinary albumin excretion were detected in women, younger subjects (age less than 60 years), overweight subjects and in those with hypertension or GFR greater than 90 (all P<0.05). In the subgroups analysis, no statistically significance of interaction term between quartiles of serum GGT level and each strata factor was detected.

## Discussion

In this community-based study, we found that serum GGT level is associated with different degrees of albuminuria. To our current knowledge, this is the largest population-based study to explore the association of serum GGT level with albuminuria. Early intervention is of great importance for chronic kidney disease, the present findings may just give insights into potential mechanism for prevention and early detection of the disease.

As a prognostic marker for nephropathy, albuminuria has been shown to correlate with structural and dysfunctional of the vasculature [23], [24]. Serum GGT level is strongly associated with vascular endothelial dysfunction in patients with advance chronic kidney disease [4]. However, only one previous study has investigated the association of serum GGT level and albuminuria. By investigating 2,478 participants from Unite States, Lee et al. [25] found that elevated serum GGT level was differently associated with the risk of micro-albuminuria (ACR: 30–299 mg/g) depending on the status of diabetes or hypertension. Results of the present study are consistent with their finding and further evidence the positive relationship between serum GGT level and macro-albuminuria (ACR: greater or equal than 300 mg/g). In addition, recent epidemiologic data suggest that low-grade albuminuria (ACR: less than 30 mg/g) have been able to predict future cardiovascular diseases [14], [15]. Therefore, we analyzed the association between serum GGT level and low-grade albuminuria and found that serum GGT level has already increased in subjects with low-grade albuminuria.

The precise mechanisms underline the association between serum GGT level and albuminuria are still not clear. Some speculations could be proposed. First, using flow-mediated dilation (FMD) as a surrogate of endothelial dysfunction, Yilmaz et al. [4] found that serum GGT level is strongly associated with abnormalities in endothelial function in patients with advance chronic kidney diseases. As an important early feature of the atherogenic process, endothelial dysfunction could therefore result in increased urinary albumin excretion [26], [27]. Second, previous epidemiological studies consistently suggest that serum GGT, even within its normal range, is an early and sensitive enzyme related to oxidative stress and inflammation [28], [29]. Cellular GGT is abundant in the kidney and could act as a protein catalyst in maintaining the degradation of glutathione [30], which is one of the major thiol antioxidant to against oxidative stress in the body [31]. In addition, serum GGT is associated with elevated C-reactive protein and involved in many pathways of inflammatory response [5], [32]. Both oxidative stress and inflammation are associated with albuminuria and might involve in the development of chronic kidney diseases [33], [34], [35]. Combined with findings of the present study, we speculated that that elevated serum GGT level might be a biomarker rather than a true causal risk factor for oxidative stress, inflammation and albuminuria.

Several limitations in this study require consideration. First, we evaluated the urinary albumin excretion on a spot morning urine sample. We admitted that the 24 hours urine or multiple samples would provide more stable results for albumin excretion [36]. However, results of spot urine samples correlate well with those of 24 hours or multiple urine samples [37], [38]. Use of spot samples for assessing urinary ACR is therefore recommended as a reliable alternative to perform in the out-patient clinic and large epidemiological specimen collection. Second, a positive but not significant relationship was detected between serum GGT level and prevalence of albuminuria in diabetes subgroup. We speculated that subjects diagnosed with diabetes were more inclined to receive further treatment, which may correct their urinary albumin excretion. Third, no causal inference can be drawn due to the cross-sectional design of the current study. Further prospective studies are needed to illustrate the precise relationship between serum GGT level and risk of albuminuria. Fourth, medication for diabetes, hypertension and dyslipidemia, especially those with angiotensin-converting enzyme inhibitors and angiotensin receptor blockers should have affected urinary albumin excretion and should be taken into account when analyzing possible risk factors associated to proteinuria. Absence of these data may influence risk estimates and result interpreting in this setting. Fifth, the results from the present study of Chinese population might not be representative of other races and younger people. The study population was predominantly female, partially because we invited residents over the age of 40 years and females are predominant in this age range in China. Additionally, an inverse association between serum GGT level and decreased eGFR was found in participants with eGFR <90 ml/min but not in those with eGFR <60 ml/min. The pathophysiological process in people with chronic kidney disease might be associated with the synthesis and deposition of GGT. Therefore, we should be cautious regarding the interpretation of whether increased GGT is a causal factor of or a consequence of decreased kidney function. Further pathopysiologic studies are needed to clarify this issue.

In conclusion, the present study demonstrates that increased serum GGT level is independently associated with prevalence of albuminuria in a large population-based cohort. Further observational studies and well-designed clinical trials are needed to be carried out to determine whether correction of serum GGT level, through lifestyle intervention or medications, could be effective to reduce the urinary albumin excretion.
